# Perianal ulcers without genital ulcers: a rare presentation of Behçet’s disease

**DOI:** 10.1093/rap/rkac047

**Published:** 2022-06-09

**Authors:** Shimpei Seiki, Yoshinobu Matsuura, Hideaki Terada, Yasuji Doi

**Affiliations:** Department of Rheumatology and General Medical, Saiseikai Senri Hospital, Osaka, Japan; Department of Rheumatology and General Medical, Saiseikai Senri Hospital, Osaka, Japan; Department of Rheumatology and General Medical, Saiseikai Senri Hospital, Osaka, Japan; Department of Rheumatology and General Medical, Saiseikai Senri Hospital, Osaka, Japan

Key message

- Perianal ulcers without genital ulcers can be a manifestation of Behçet’s disease.

Dear Editor, Behçet’s disease (BD) is a systemic immune-mediated disease characterized by recurrent oral ulceration, genital ulceration and skin and ocular lesions. Among these, oral ulceration is the most frequent (93%), followed by skin lesions (81%), genital ulceration (63%) and ocular lesions (37%) [1]. Although the frequency of gastrointestinal (GI) lesions in patients with BD ranges from 5 to 25% in East Asia, anal complications are considered an unusual GI manifestation [2]. Herein, we present a rare case of a patient with BD who had perianal ulcers without genital ulcers.

A 48-year-old Japanese woman was admitted to our hospital with a fever, oral aphtha, skin rash, arthralgia, myalgia and anal pain. She had been suffering from recurrent oral aphthae since adolescence. Approximately 1 month before admission, she presented with gastric discomfort and underwent an upper endoscopy and colonoscopy, which revealed no specific findings except for gastro-oesophageal reflux and a colonic polyp. The symptoms resolved spontaneously within a month. Nine days before admission, she developed a fever (temperature >38°C) and right shoulder and knee arthralgia, followed by skin rashes, left thigh myalgia and anal pain. She had no other history, family history, medication or complications.

On admission, she had multiple deep oral aphthae with severe pain on the tongue, gingiva and buccal mucosa. In addition, erythematous papules, pustules and small nodules with tenderness were observed on her face, neck, trunk and limbs (Fig. 1A and B). She presented with a new-onset perianal ulceration, although gynaecological examination did not reveal any genital ulceration (Fig. 1C). An ophthalmological examination revealed no ocular lesions. No gastrointestinal symptoms, such as abdominal pain, diarrhoea or bloody stools, were observed.

**Fig. 1:**
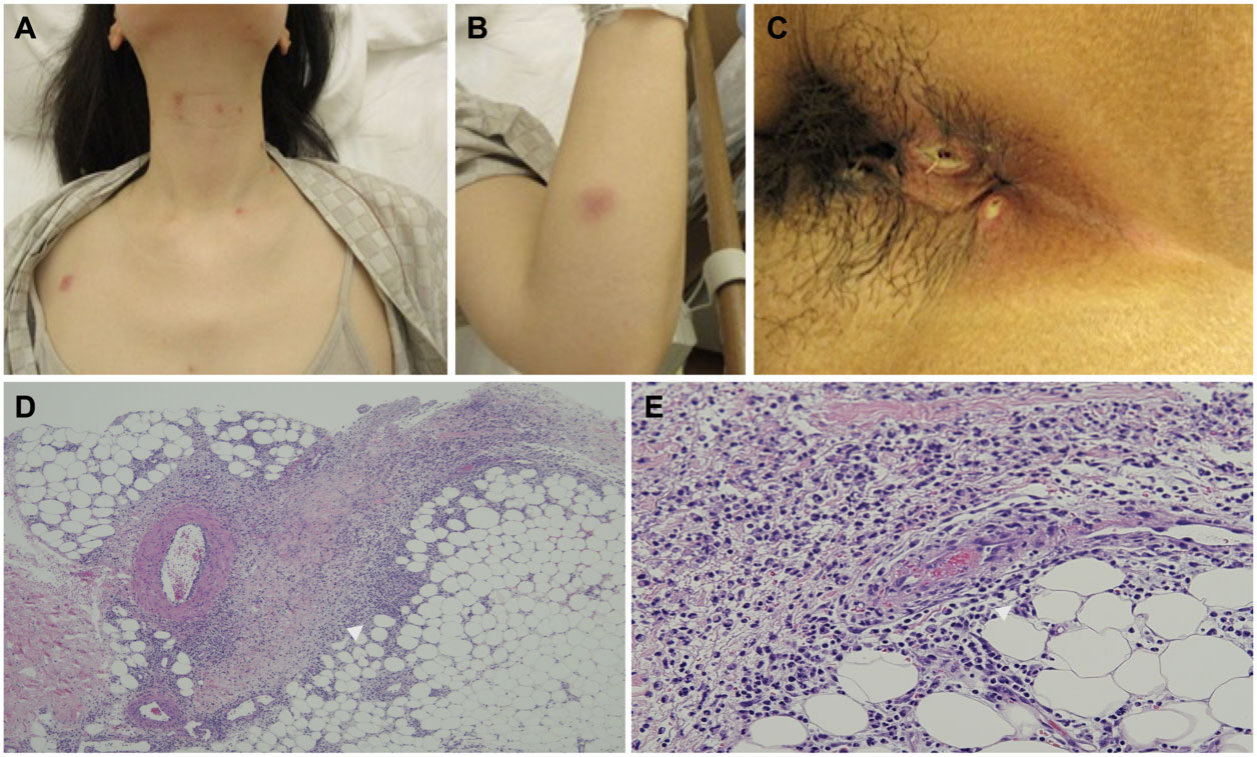
Clinical manifestations and pathological findings (**A**) Erythematous papulae on the neck and trunk. (**B**) Erythematous small nodule in the left arm. (**C**) Perianal ulcerative lesions. (**D**) Microscopic examination of the erythema revealed septal panniculitis with thickened lobular septa and prominent infiltration of neutrophils (white arrowhead), indicating hallmark features of the erythema nodosum-like lesion in Behcet’s disease (Haematoxylin and Eosin stain, ×40). (**E**) Thrombophlebitis in the superficial cutaneous veins (white arrow): a finding consistent with Behcet’s disease (Haematoxylin and Eosin stain, ×400).

Chest and abdominal CT showed no significant findings suggestive of fever. Blood test results were as follows: CRP, 19.7 mg/dl; white blood cell count, 12.4 × 10^3^/µl; and increased ESR (42 mm/h). The patient tested negative for ANCA and ANA. Urinalysis revealed no abnormalities. Blood culture and serum syphilis test results were negative, and culture and cytology of the anal ulcers did not reveal any specific findings. Biopsy of the skin sample obtained from the erythematous left leg revealed neutrophil and mononuclear cell infiltration into the subcutaneous adipose tissue and capillaries, suggesting erythema nodosum-like histology, as seen in BD (Fig. 1D). Furthermore, pathological findings showed fresh thrombus formation in the veins, indicating thrombophlebitis (Fig. 1E). The patient was HLA-A24 and -B51 positive. She was eventually diagnosed with BD because she fulfilled the International Criteria for BD [3].

Treatment was initiated with 100 mg methylprednisolone for 3 days, followed by 30 mg oral prednisolone administered concomitantly with colchicine. Hydrocortisone ointment was applied to the perianal ulcer. The fever resolved on day 2 after starting methylprednisolone administration, and systemic symptoms, including myalgia, skin rash, oral ulceration, arthralgia and perianal ulceration, improved rapidly; all symptoms disappeared on day 10. Thereafter, prednisolone was tapered and eventually discontinued, with no relapse for 3 months.

This case is unique in two respects: first, the patient presented with only perianal ulcers and without other GI lesions; and second, without genital ulcers. BD-associated GI lesions can appear from the oesophagus to the anus; nonetheless, ileocaecal lesions are more common, occurring in nearly 70% of cases [4]. Unlike Crohn’s disease, anal lesions (including anal fissures and fistulae) are observed in <1% of BD cases. Although there are a few reported cases of BD with anal complications similar to those seen in Crohn’s disease (such as refractory anal fissures and fistulae), the present case, with only perianal ulcers, seems distinct from these cases [5, 6]. Moreover, other accompanying GI lesions have been reported in previous cases, whereas the present patient had only anal lesions.

Conversely, Iwama and Utzunomiya [7] reported a case of BD in which a patient had perianal ulcers without anal fissures, fistulae or other GI lesions. In that patient, glans penis ulceration was observed, whereas our patient did not exhibit any genital ulcers, making diagnosis difficult. According to a report on genital ulcers in 207 patients with BD, only 8 and 4% of 137 women presented with ulcers in the perineal and perianal regions, respectively, which are considered atypical locations [8].

Although the time from onset to diagnosis of BD is long, some patients do not meet the diagnostic criteria despite the presence of severe organ involvement, such as intestinal lesions. In patients with anal lesions only, as in the present case, BD is not likely to be suspected. Given that perianal ulcers can be present without other GI lesions or genital ulcers, the possibility of BD must be considered during the diagnosis and follow-up of such patients.

*Funding:* No specific funding was received from anybody in the public, commercial or not-for-profit sectors to carry out the work described in this article.

*Disclosure statement:* The authors have declared no conflicts of interest.

*Consent:* Informed consent was provided for the publication of this paper.

## Data availability statement

Data sharing is not applicable to this article because no new data were created or analysed in this study.
